# Re: Persistent Mullerian Duct Syndrome: a rare entity with a rare presentation in need of multidisciplinary management

**DOI:** 10.1590/S1677-5538.IBJU.2017.0072

**Published:** 2017

**Authors:** Lisieux Eyer de Jesus

**Affiliations:** 1 Departmento Cirurgia Pediátrica, Hospital Universitario Antonio Pedro, Niterói, RJ, Brasil

*To the editor,*

We would like to make some comments about the article “Persistent mullerian duct syndrome: a rare entity with a rare presentation in need of multidisciplinary management” (1).

The authors report a DSD case diagnosed as PMDS and review some clinical aspects of this exceedingly rare syndrome, but some unclear aspects are notable. The patient described seems to be a case of ambiguous genitalia (proximal hypospadias and bilateral cryptorchidism), classified as 46,XY DSD. No gonadal biopsy was done and only the right gonad was found/described. Anti-mullerian hormone dosages are not available.

In most PMDS cases the patient presents normal testes or testicles only secondarily affected by cryptorchidism. Primary testicular failure, as seen in this patient, has never been previously described. To the best of our knowledge no PMDS cases associated to streak gonads, dysplastic gonads or absent gonads have been described till this moment, despite anatomically complicated cases of cryptorchidism being common, including crossed testicular ectopy.

Also, in PMDS the external genitalia is of normal male (see Table-1). The only known exception to this moment is the patient described in reference 12, but in his case two normal cryptorchidic “peeping” testes were found.

From our point of view other DSD diagnoses are still possible, especially mixed gonadal dysgenesis, that may associate to a variety of karyotypes. Abnormal/absent gonads, primary and precocious testicular failure and the absence of a Fallopian tube at the left side are also compatible with this alternative diagnosis (in PMDS cases the uterus is anatomically normal, despite being atrophic).
